# Infant Formula Supplemented with Biotics: Current Knowledge and Future Perspectives

**DOI:** 10.3390/nu12071952

**Published:** 2020-06-30

**Authors:** Seppo Salminen, Bernd Stahl, Gabriel Vinderola, Hania Szajewska

**Affiliations:** 1 Functional Foods Forum, Faculty of Medicine, University of Turku, 20520 Turku, Finland; seppo.salminen@utu.fi; 2 Danone Nutricia Research, 3584 CT Utrecht, The Netherlands; bernd.stahl@danone.com; 3 Department of Chemical Biology & Drug Discovery, Utrecht Institute for Pharmaceutical Sciences, Utrecht University, 3584 CG Utrecht, The Netherlands; 4 Instituto de Lactología Industrial (INLAIN, UNL-CONICET), Facultad de Ingeniería Química, Universidad Nacional del Litoral, Santiago del Estero 2829, Santa Fe 3000, Argentina; gvinde@fiq.unl.edu.ar; 5 Department of Paediatrics at the Medical University of Warsaw, 02091 Warsaw, Poland

**Keywords:** human milk oligosaccharides, probiotics, prebiotics, synbiotics, postbiotics, 2′-fucosyllactose (2′-FL), lacto-N-neotetraose (LNnT), 3′-galactosyllactose (3′-GL), breastfeeding, infant formula

## Abstract

Breastfeeding is natural and the optimal basis of infant nutrition and development, with many benefits for maternal health. Human milk is a dynamic fluid fulfilling an infant’s specific nutritional requirements and guiding the growth, developmental, and physiological processes of the infant. Human milk is considered unique in composition, and it is influenced by several factors, such as maternal diet and health, body composition, and geographic region. Human milk stands as a model for infant formula providing nutritional solutions for infants not able to receive enough mother’s milk. Infant formulas aim to mimic the composition and functionality of human milk by providing ingredients reflecting those of the latest human milk insights, such as oligosaccharides, bacteria, and bacterial metabolites. The objective of this narrative review is to discuss the most recent developments in infant formula with a special focus on human milk oligosaccharides and postbiotics.

## 1. Introduction

Nutrition in early infancy and childhood can significantly impact growth and development as well as immediate and later health [1]. The World Health Organization recommends exclusive breastfeeding for the first six months of life, followed by continued breastfeeding with appropriate complementary foods for up to two years or beyond [2]. Breastfeeding and/or nutritional intervention during early life can help prevent both infectious and non-communicable disease risk during childhood and into adulthood [1]. 

A healthy gut development is of major importance during infancy. It contributes to growth and development by ensuring digestion and absorption of nutrients and fluids. The gut is also key in the development of immunity insofar as it maintains a barrier against infectious agents and interacts directly with the immune system to induce mucosal and systemic tolerance, which prevents of allergy. Furthermore, the gut provides signals to the brain to maintain a healthy state [3].

One of the major factors enabling proper gut function and development is a balanced gut microbiota [4]. Several prenatal and perinatal factors including mode of delivery, use of antibiotics, diet, and other environmental factors, including geographic region, may influence the microbial colonisation of the infant and in turn the maturation of the immune system, as reviewed elsewhere [5]. Hence, it is generally agreed that the gut microbiota of the healthy, full-term, vaginally delivered and breastfed infant constitutes the gold standard for a favourable microbial composition in early life [6]. Since human milk contributes remarkably to the development of a balanced gut microbiota—amongst many other health benefits—it is essential for infant formula to be as close to human milk as possible, providing bioactives targeting gut and immune health [7].

Two human milk oligosaccharides (HMOs), 2′-fucosyllactose (2′FL) and lacto-N-neotetraose (LNnT), are recent examples of optional ingredients being added to infant formula [8,9,10,11]. However, little attention has yet been given to 3′-galactosyllactose (3′-GL), which is an HMO present in human milk that may also naturally occur in fermented infant formula derived by milk fermentation [12,13]. This narrative review will focus on these most recent developments in infant formula as examples of ‘biotics’ aiming to resemble human milk functions in infant formula.

## 2. Oligosaccharides, Bacteria, and Microbial Metabolites in Human Milk

Human milk contains many bioactive compounds such as oligosaccharides, immune cells, and varying levels of bacteria and their metabolites. They play an important role in the development of a healthy gut by supporting a favourable intestinal microbiota and in the development of the infant’s immune system [14,15,16,17,18].

### 2.1. Human Milk Oligosaccharides (HMOs)

HMOs are pools of complex carbohydrates, the third most abundant component of human milk, and one example of naturally occurring ‘prebiotics’ [19]. Although originally characterised as HMOs, they are also present in the amniotic fluid, and thus the foetus is already exposed to HMOs prior to birth when ingesting the amniotic fluid [20,21,22,23]. 

The quantity of HMOs in mature human milk is approximately 12–15 g/L as reported elsewhere [24,25]. There are more than 200 structurally different HMOs working together to deliver a combined beneficial effect, of which today more than 160 (neutral and acidic) types have been characterized in detail [26]. 

Most HMOs escape digestion in the small intestine [16] and progress to the colon acting as decoy molecules binding pathogens and getting metabolised as ‘food’ for the commensal gut bacteria [27], such as bifidobacteria and lactic acid bacteria, allowing such bacterial populations to become more abundant [28]. Besides their prebiotic effect, HMOs also have direct effects on immune cells [29,30,31,32], block the routes of infections [30,33], provide building blocks for the brain [34], and stimulate intestine barrier functions, as published elsewhere [35].

**Figure 1:**
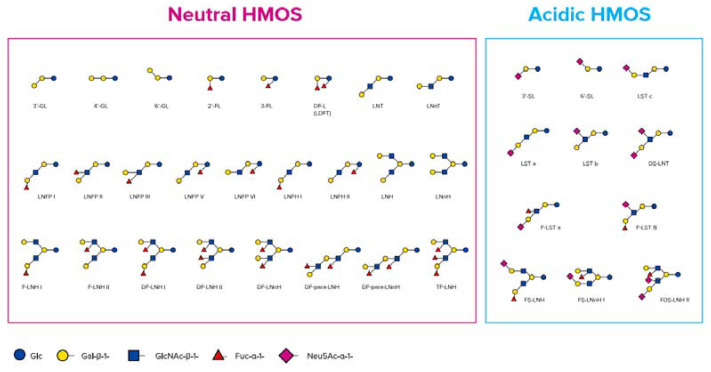
Chemical structure of neutral and acidic human milk oligosaccharides (HMOs) (adapted from Thurl et al. [25], Newburg et al. [38], and Urashima et al. [26]).

HMOs are synthesised in the mammary gland by the prolongation of lactose with monosaccharides forming non-digestible trisaccharides (DP3) (degree of polymerisation, which is the amount of monosaccharide building blocks) or tetrasaccharides (DP4). The addition of sialic acids leads to two different sialyllactoses (SL), 3′-SL and 6’-SL (DP3), and the addition of fucose leads to two different fucosyllactoses (FL), 2′-FL and 3-FL (DP3), as well as difucosyllactose (DP4) [25,36,37] (Figure 1). 

**Figure 2:**
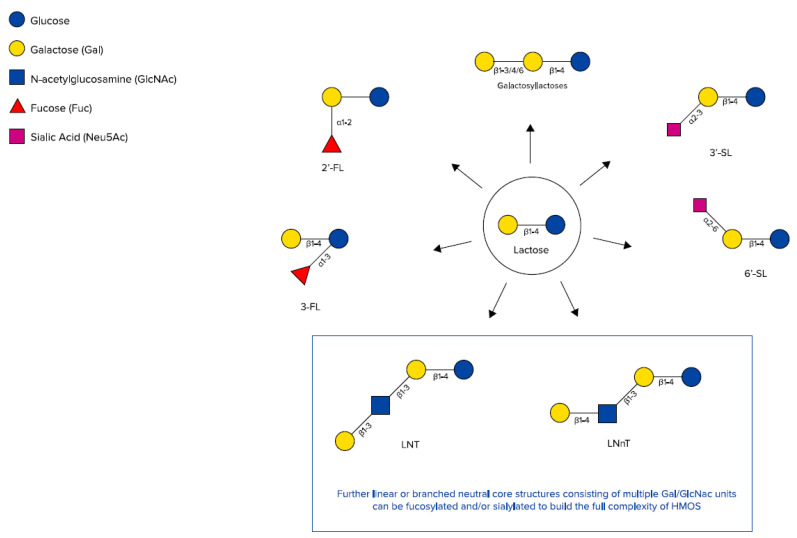
Chemical structures of galactosyllactoses, 2′-FL, 3-FL, 3′-SL, 6’-SL, Lacto-N-tetraose (LNT), and lacto-N-neotetraose (LNnT) (adapted from Urashima et al. [48]).

HMOs also include galactosyllactoses (GLs), which appear in the form of several structurally distinct isomers and only differing in the glycosidic linkage of the terminal galactose added to lactose, leading to 3′-GL, 4’-GL, and 6‘-GL (DP3) [38,39] (Figure 2). Those HMOs can also be derived from lactose by the enzymatic activity of bacterial β-galactosidases, which splits lactose to glucose and galactose and catalyses the transgalactosylation of lactose to produce GLs [40,41,42,43]. Using an adapted approach of a recently published targeted liquid chromatography-tandem mass spectrometry method for HMOs, native GL-isomers like 3′-GL could be directly detected in human milk samples from various stages of lactation. The abundance of 3′-GL appeared to be relatively stable between colostrum and mature milks, whereas 6’-GL declined over time [44]. In general, HMO concentrations decline over time from colostrum to mature milk [45], including GLs [39,46]. Thurl et al. also found a slight decrease in HMOs within the first 90 days of lactation [47]; however, this might be partly compensated for by more volume of human milk being consumed by infants in the respective longitudinal setting.

Larger HMO structures are derived by adding galactose and N-actetylglucosamine as disaccharide building blocks, which leads to tetrasaccharides (DP4), Lacto-N-tetraose (LNT), and Lacto-N-neotetraose (LNnT). Those tetrasaccharides can be further extended by those disaccharide units (DP6, DP8, etc.) and in addition decorated with further single or multiple fucose residues, leading to large neutral HMOs with a molecular size up to 8 kDa [49] and/or single or multiple sialic acid residues, leading to large acidic HMOs with a molecular size up to 3.6 kDa [50]. 

Size exclusion chromatography, which was further used to describe the complexity of short- and long-chain HMOs, demonstrated a molecular size distribution of short-chain and long-chain HMOs in a ratio of 9:1 [49]. Long-chain HMOs are less easily fermented by the gut microbiota, whereas short-chain HMOs are more active in the proximal parts of the gut [26], ensuring that potential substrates are available for the gut microbiota through the gastrointestinal tract, as shown for homo-polymeric fructans [51]. Although the long-chain HMOs are less abundant, they may exert a relevant bioactivity, which leads to the conclusion that abundance cannot automatically explain the biological importance of a molecule.

### 2.2. Bacteria in Human Milk

Human milk is also an important source of beneficial bacteria (naturally occurring ‘probiotics’) that help colonise the infant gut and contribute to the composition of a favourable gut microbiota, including *Bifidobacterium* spp. and *Lactobacillus* spp. [52,53], with genus *Bifidobacterium* dominating the gut microbiota of a vaginally delivered infant [54,55]. Bacteria in human milk are anticipated to be bioactive components regulating the development of an infant’s immune system and attenuating inflammation processes [56]. In general, the microbiome of human milk is a recent field of research, as explored elsewhere [53,56,57,58,59].

The total number of bacteria in human milk significantly differs according to detection methods. It has been estimated that human milk contains median values between 10^3^ and 10^6^ bacteria per millilitre [60,61,62]. The difference in number caused by different detection methods could be due to the fact that in molecular-based methods, DNA from non-viable bacteria and extracellular DNA can also be amplified, suggesting that not only live bacteria but significant amounts of non-viable bacteria coexist in human milk [60].

### 2.3. Microbial Metabolites in Human Milk

Besides bacteria, their metabolites (e.g., butyrate and other short-chain fatty acids (SCFAs), peptides, oligosaccharides) may also naturally pass into human milk, and this can be detected through metabolomics research using nuclear magnetic resonance spectroscopy [63,64]. Most recently these metabolites have gained more research attention that is focused on their possible role in shaping the growth and development of the infant. A widely accepted definition still needs to be agreed on, but in general, they may also be referred to as ‘natural postbiotics’ (comprising both inactivated bacterial cells and metabolites) and are anticipated to stimulate both healthy gut microbiota composition and function as well as immune functioning and development [60,64,65]. 

## 3. The ‘Biotic’ Family: Resembling Human Milk Benefits in Infant Formula

**Figure 3:**
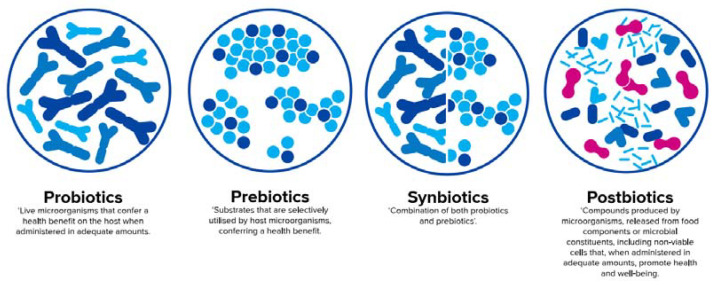
Definition of probiotics [69], prebiotics [70], synbiotics [71], and postbiotics [65] (adapted from Salminen et al. [72]).

There is increasing research on nutritional postnatal interventions using probiotics, prebiotics, synbiotics, and the so-called postbiotics to promote the establishment of a beneficial microbiota and to have a positive impact on neonatal health (Figure 3). Whereas infant formulas can only provide static selections of these substances, the amount and composition are highly dynamic and individual in human milk [47,66,67]. This may be illustrated by the diversity of oligosaccharide structures in human milk, which has been shown to vary among women according to genetic factors, geographical regions, stages of lactation, and, potentially, maternal probiotic supplementation during the late stages of pregnancy [45,47,68]. For example, in women with the active gene for fucosyltransferase 2 (FUT2, secretors), 2′-FL is by far the most abundant HMO, with a mean concentration of 2.7 g/L, and constitutes nearly 24% of all HMOs [25]. However, in the milk of women without the active gene for FUT2 (non-secretors), who make up 21% of all women in larger parts of the world, 2′-FL was not found [24,25,67]. 

According to a recent consensus definition, probiotics are live microorganisms that confer a health benefit on the host when administered in adequate amounts [69], whereas prebiotics are defined as substrates that are selectively utilised by host microorganisms conferring a health benefit [70]. An official definition of synbiotics, which are a combination of both pro- and prebiotics [71], will soon be published by the International Scientific Association for Probiotics and Prebiotics (ISAPP). In contrast to pre- and probiotics, an official aligned global definition of postbiotics is still pending and will be discussed later in this paper.

### 3.1. Probiotics

Most probiotic-containing infant formula comprise *Bifidobacterium* spp. and/or lactic acid bacteria such as *Lactobacillus* spp., which are generally regarded as safe for food use in the European Union based on the QPS-list (Qualitative Presumption of Safety) of bacteria. In the United States, a non-mandatory system of safety evaluation is in place, provided by the U.S. Food & Drug Administration (FDA). On request, it evaluates safety assessment filings of specific probiotic strains to be included in the so-called GRAS (Generally Recognized as Safe) Notice Inventory, which is continuously updated [73]. 

There are data on specific probiotics in infant formula accompanied by a large diversity in study outcomes [74]. However, these health benefits are very strain and disease specific. For example, for preterm infants, the Committee on Nutrition of the European Society for Paediatric Gastroenterology and Nutrition (ESPGHAN) and the ESPGHAN Working Group for Probiotics and Prebiotics conditionally recommended the use of *L. rhamnosus* GG (LGG) ATCC 53103 (at a daily dose ranging from 1 × 10^9^ CFU to 6 × 10^9^ CFU) and the combination of *B. infantis* Bb-02, *B. lactis* Bb-12, and *Str. thermophilus* TH-4 (at a daily dose of 3.0 to 3.5 × 10^8^ CFU of each strain) as it might reduce necrotizing enterocolitis (NEC) stage 2 or 3 in preterm infants (low certainty of evidence), but mortality and sepsis did not show any clear direction in effect size. However, no recommendation could be made in either direction regarding the use of *L. reuteri* DSM 17938 and the combination of *B. bifidum* NCDO 1453 (currently reclassified as *B. longum*) with *L. acidophilus* NCDO 1748 (ATCC 4356, LA37, or NCIMB 30316) in preterm infants to reduce the risk of mortality, NEC stage 2 or 3, or sepsis (very low certainty of evidence) [75]. For term born infants, in a review from 2011, the ESPGHAN Committee on Nutrition did not recommend the routine use of probiotic- and/or prebiotic-supplemented infant formulas, identifying further need for well-designed and carefully conducted randomised controlled trials [74].

### 3.2. Prebiotics

Because of their stability, low risk of adverse effects, ease of administration, and potential for influencing the composition and function of the microbiota in the gut and beyond, the clinical applications of prebiotics are expanding [76]. 

In last decades, different prebiotic mixtures of galacto-oligosaccharides (GOS) and fructo-oligosaccharides (FOS) have been studied. Globally the most studied prebiotic mixture of oligosaccharides in infant formula consists of short-chain (sc) GOS and long-chain (lc) FOS, scGOS/lcFOS (9:1). The mix is in the range of HMOs close to human milk in quantity (8g/L) and diversity (more than 100 different structures of short- and long-chain types in a ratio of 9:1). Although this prebiotic mixture approaches the molecule size distribution of short- and long-chain oligosaccharides in human milk, it is not structurally similar to HMOs [77]. Clinical research on scGOS/lcFOS (9:1) showed benefits linked to modulation of the gut microbiota and the immune system, reduced incidence of infections, and stool softening [76,78,79]. This prebiotic mixture has been recognised as a prebiotic by ISAPP [70], and it may be added to infant formula and follow-on formula according to the Commission Delegated Regulation (EU) 2016/127 [80]. However, to date, there are no health claims granted by the European Food Safety Authority (EFSA) for the application of any oligosaccharides in infant formula.

In addition to prebiotics based on GOS and/or FOS, infant formulas have recently been supplemented with specific technically derived HMOs (e.g., 2′-FL and LNnT), which have been anticipated as candidate prebiotics [70] (see Section 3.3).

### 3.3. Specific HMOs Added to and/or Present in Infant Formula

Recent research highlights the variety of HMOs in human milk, underlining the importance of scientific assessment of the role of both minor and major oligosaccharides in promoting infant health before adding it to infant formula. 

Technically, only 2′-FL and LNnT are currently commercially available for addition to infant formula as purified ingredients [81]. The technology used for obtaining these is the coupling of bacterial homogenates or the utilisation of *E. coli* as a microbial cell factory (see Table 1). Other technologies are available but, currently, only used for analytical and research purposes. While 2′-FL and LNnT are obtained by chemical synthesis, 3′-GL occurs as a bacterial by-product during milk fermentation [12,13]. Besides these three examples, further HMOs are in the pipeline for application in infant formula [81].

#### 3.3.1. HMOs 2′-FL and LNnT

2′-FL and LNnT have been anticipated as candidate prebiotics [70]. Although manufactured HMOs are structurally identical to their counterparts in human milk, regulatory approval is required for novel foods by the European Union (Commission Implemented Regulation (EU) 2017/2470) [93]. The EFSA Scientific Panel on Nutrition recently assessed 2′-FL, difucosyllactose (DFL), LNnT, and LNT as safe for use in infant formula [94,95,96]. Furthermore, 2′-FL, DFL, LNnT, and LNT have been added to the GRAS-list derived from the FDA [97]. 

Preclinical research has shown that synthetic 2′-FL (with an identical structure as the 2′-FL found in human milk and often prepared from lactose) has prebiotic effects and may deliver functional benefits in infants. In preclinical trials, 2′-FL promoted the growth of specific bifidobacteria [98,99], blocked the growth of pathogens [100,101,102], and supported gut maturation and the stimulation of the gut intestinal barrier [103]. Furthermore, 2′-FL impacted neuronal dependent gut migrating motor complexes, suggesting beneficial effects on the central nervous system [104].

Infant formulas containing 2.4 g total oligosaccharides/L (control: scGOS only; experimental formulas: scGOS + 0.2 or 1.0 g 2′-FL/L) have been demonstrated to be safe in terms of normal growth and were well-tolerated in a prospective, randomised, an controlled growth and tolerance study with 189 healthy, singleton infants [9]. A post-hoc analysis of this study revealed that infants receiving 0.2 g/L 2′-FL had a significantly reduced incidence of respiratory infections as compared to the control group receiving scGOS alone. This effect was not seen in the group receiving 1.0 g/L 2′-FL [10]. A further analysis of the same study showed that infants who received the 2′-FL-containing formulas had significantly lower plasma inflammatory cytokines compared to infants who received the control formula, closely resembling the levels found in the breastfed reference group, potentially indicative of an anti-inflammatory effect [8].

A recently developed infant formula concept combines a prebiotic mixture of scGOS/lcFOS (9:1) and 2′-FL to further mimic the complex composition of HMO structures and their functional benefits. Data from the first in vivo pre-clinical investigations showed positive effects of this combination in a rotavirus model [105] and in a vaccination model [32,106].

LNnT has been assessed in vitro for function as a prebiotic inducing growth and metabolic activity in *Bifidobacterium infantis* [107]. As an ingredient, LNnT proved to be a stable and safe component of infant formula that was evaluated for its ability to reduce oropharyngeal colonisation with *Streptococcus pneumoniae* in children (6 months or older). Although colonisation was not reduced, LNnT proved to be well-tolerated at the tested concentration of 200 mg/L [107]. 

Infant formula supplemented with a combination of 2′-FL (1.0 g/L) and LNnT (0.5 g/L) was demonstrated to be safe and well-tolerated and to support age-appropriate growth in a multicentre, randomised, double-blind trial with 175 healthy infants, with weight gain as a primary study outcome. Secondary outcomes were also found including associations of lower parent-reported morbidity (particularly bronchitis) and medication use (antipyretics and antibiotics) in infants fed the supplemented formula compared to the control [11].

#### 3.3.2. HMO 3′-GL

HMO 3′-GL is naturally present in human milk and was already isolated from human milk in 1988 [40]. Recently, a single chromatography run using an improved sample pre-treatment method and ultrahigh pressure liquid chromatography with fluorescence detection quantified 15 sialylated and neutral HMOs with high sensitivity, identifying 3′-GL and 6’-GL in colostrum, transitional, and mature human milk [108]. Human milk from mothers with preterm delivery revealed 3′-GL concentrations of 4–28.82 µg/mL (median 10.44) at 1 w postpartum, whereas concentrations ranged from 4–32.97 µg/mL (median 12.34) in colostrum and 4–20.73 µg/mL (median 4) at 8 w postpartum from mothers with term delivery [46]. Further human milk analyses of mothers with term delivery revealed 47–79 µg/mL of 3′-GL in colostrum [109], 5.08 ± 0.45 µg/mL (mean ± SD) in colostrum, and 4.84 ± 0.48 µg/mL (mean ± SD) at 100 d of lactation [39], and 0.5–39 µg/mL (median 4.6) in milk pooled until 21 d of lactation [38].

In pre-clinical studies, 3′-GL, 4’-GL, and 6’-GL prepared from colostrum individually accounted for specific immunomodulation of polyinosinic:polycytodylic acid-induced interleucin-8 levels in an immature human intestine tested at a concentration of 200 µg/mL each [109]. Another study reported that a solution of 5 mg galactosyloligosaccharides/mL, which was synthesized from lactose and comprised of 14 % 3′-GL, 8 % 4’-GL, and 12 % 6’-GL, demonstrated ex vivo anti-inflammatory effects by attenuating the nuclear transcription factor κB inflammatory signalling in human intestinal epithelial cells [38]. In a model for intestinal barrier function using human intestinal epithelial Caco-2 cell monolayers grown in a transwell system, 3′-GL chemically synthesised from lactose was able to protect the intestinal barrier against breakdown of intestinal integrity, whereas alpha-3′-GL (with an α1-3 glycosidic linkage), 4’-GL, and 6’-GL (all chemically synthesized from lactose) demonstrated no significant results [110]. Previously, a commercial mixture of galacto-oligosaccharides also demonstrated a barrier-stabilising effect and resulted in improved integrity of the intestinal barrier function and reduced inflammatory response by using the same in vitro method [111].

Although these first pre-clinical data may indicate that certain galactosyllactoses, such as 3′-GL, have protective and immunomodulatory effects in the gut, more research is needed to further explore their role as HMOs.

Since 3′-GL in infant formula is a fermentation by-product, the safety of 3′-GL can be considered assured by the long history of safe use of the fermented formula in France [112]. The clinical evidence available on fermented infant formula is summarized in Section 3.5.3. 

### 3.4. Synbiotics

Beneficial synergistic effects may be expected from a combination of probiotics and prebiotics, which are called synbiotics, using prebiotics to selectively increase abundance of both endogenous beneficial bacteria and beneficial microbes in the infant gut [113].

Given the evidence for an aberrant gut microbiota in infants with allergy [114,115] and the key role of the gut microbiota on immune system maturation [116], there is a strong rationale for developing a suitable pre- and probiotic (synbiotic) blend for use in infant formula for infants at high risk of allergy and infants with already developed allergy. A synbiotic mixture of prebiotics (scGOS/lcFOS or scFOS/lcFOS in a ratio of 9:1) and the probiotic strain *Bifidobacterium breve* M-16V already demonstrated promising results. This synbiotic combination compensated for delayed bifidobacteria colonisation in infants delivered by caesarean section (compared to vaginally delivered infants) in an exploratory, randomised, double-blind, controlled study with 153 infants with detection of total faecal bifidobacteria as primary study outcome [117]. Secondary outcomes demonstrated a lower proportion of potential pathogens (e.g., clostridia-related species) [118], lower faecal pH, and significant changes in SCFA pattern (e.g., higher acetate and lower butyric, isobutyric, and isovaleric acids) [119]. Furthermore, this synbiotic blend led to a significant decrease of SCORAD (Scoring Atopic Dermatitis) score in infants with atopic dermatitis (AD) and greater improvement of SCORAD score in infants with IgE (immunoglobulin E)-associated AD [119]. At the 1-year follow-up of this study, children with AD in the synbiotics group showed less asthma-like symptoms (frequent wheezing and wheezing and/or noisy breathing apart from colds) and asthma medication use [120]. 

However, even if some synbiotic combinations already display promising clinical results in infants, more randomised trials with longer follow-up are needed for the determination of their physiological and metabolic impact on the host [113].

### 3.5. Postbiotics

In contrast to probiotics, bacterial viability in postbiotics is not seen as an essential requirement for health benefits, providing a potential opportunity to foods that are not convenient for carrying viable bacteria [121], e.g., liquid infant formula.

In infant formulas, the concept of postbiotics is yet to be defined, although specific fermented infant formulas with postbiotics have been commercially available in Europe for decades. So far, most known postbiotics are derived from *Lactobacillus* and *Bifidobacterium* strains, which are also generally the most used probiotics [65].

#### 3.5.1. Definition of Postbiotics

Recently, a provisional definition has been formulated that stipulates that postbiotics are compounds produced by microorganisms and released from food components or microbial constituents, including non-viable cells that, when administered in adequate amounts, promote health and well-being [65]. 

Examples of postbiotics have been summarised as follows [65]:

- Compounds deriving from bacterial metabolism, such as exopolysaccharides, vitamins, lactic acid, bacteriocins, enzymes, surfactants, antioxidants, and SCFAs.
- Complex molecules released from food compounds (enzymatically produced during food fermentation), such as peptides and galacto-oligosaccharides, e.g., 3′-GL and 6‘-GL.
- Components released from lysed cells including DNA, RNA, cell walls and, perhaps, other cytoplasmic components, and surface layer proteins.

A consensus definition on postbiotics is currently being formulated by ISAPP [122].

#### 3.5.2. Postbiotics through Fermentation

For many thousands of years, fermentation of food has been applied as a natural process to generate foods with particular properties, palatability, taste, and health benefits [113]. Most health benefits of fermented functional foods are accomplished by either the live microorganisms ingested or by postbiotics deriving from these microorganisms [65]. 

For cow’s milk-based infant formulas, fermentation processes typically use lactic acid-producing bacteria as a starter culture [65]. In addition to lactose consumption during fermentation, microbial enzymatic transgalactosylation can transform lactose into other lactose-based biomolecules, with 3′-GL as the major trisaccharide produced by *Streptococcus thermophilus* (e.g., *Streptococcus thermophilus* 065) from lactose fermentation [13]. The fermentation process is usually followed by physical treatment, which may include homogenisation, pasteurisation, sterilisation, and/or spray-drying [123]. 

Recently, a specific fermentation process has been developed for infant nutrition, using two specific types of food-grade lactic acid producing microorganisms (*Bifidobacterium breve* C50 and *Streptococcus thermophilus* 065) and naturally delivering postbiotics [12,124,125].

#### 3.5.3. Benefits of Postbiotics in Infant Formula

**Figure 4:**
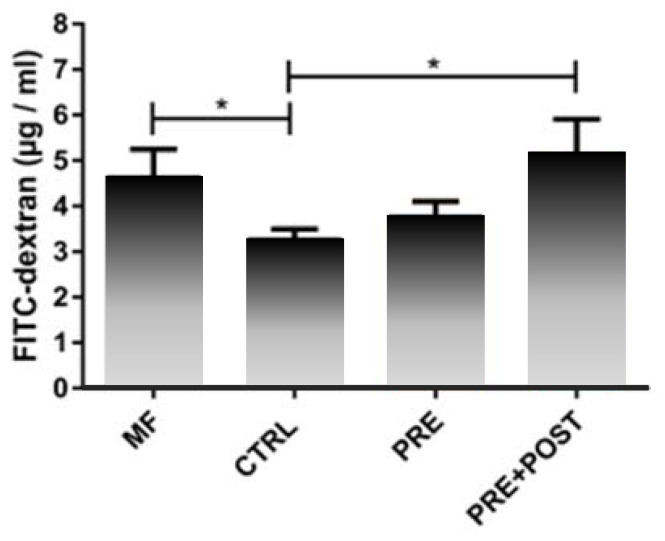
Gut permeability measured by fluorescein isothiocyanate (FITC)-dextran in mice fed infant formula (IF) containing postbiotics deriving from a specific fermentation process (Lactofidus^TM^) and prebiotic scGOS/lcFOS (9:1) (PRE+POST) compared to IF with prebiotics only (PRE), control IF without post- and prebiotics (CTRL), and mother-fed mice (MF); data represent mean + standard error of the mean (SEM); one-way ANOVA + post-test; *p ≤ 0.05 [126]. Results derive from a congress abstract and are currently in preparation for full publication. Courtesy of Dr A. Vincent, Dr I. Renes and Dr I. Van Seuningen.

In a mouse model, infant formula containing postbiotics deriving from a specific fermentation process combined with prebiotic scGOS/lcFOS (9:1) stimulated morphological (i.e., crypt villus length in ileum) and functional (i.e., ileal sucrase activity, gut permeability) gut maturation more similar to the mother-fed situation than infant formula without pre- or postbiotics [126]. Gut permeability measured by fluorescein isothiocyanate-dextran was similar in mice receiving post- and prebiotics and mother-fed mice, while it was significantly lower in mice receiving the control infant formula without post- or prebiotics (Figure 4). An early decrease in permeability, as observed in the control group, could have a lasting detrimental effect on health by changing immune maturation [127].

The first clinical study with a fermented infant formula was already published in 1989, reporting positive effects on gut function [128]. More recently, specific postbiotics deriving from fermentation have been reported to positively affect the gut microbiota, metabolic pathways, and immune responses [4,129,130]. 

Specifically, an infant formula fermented by *Bifidobacterium breve* C50 and *Streptococcus thermophilus* 065—with and without prebiotic scGOS/lcFOS (9:1)—demonstrated its capacity to impact immune and gut health benefits in otherwise healthy infants (Table 2). Since fermented formulas may consist of a variety of bacterial metabolites, their benefits cannot be attributed to any single metabolite alone.

In a recent systematic review, limited evidence was demonstrated for specific postbiotics being recommended for treating paediatric diarrhoea and preventing common infectious diseases among children. For therapeutic trials, supplementation with heat-inactivated *Lactobacillus acidophilus* LB reduced the duration of diarrhoea, and for preventive trials, heat-inactivated *Lactobacillus paracasei* CBA L74 reduced the risk of diarrhoea, pharyngitis, and laryngitis [131].

In view of the suggested preclinical and clinical results, postbiotics should be considered as strain-specific as probiotics: each bacterial strain and each fermentation process generate unique cells, cell structures, and metabolites with varying functionalities, and the benefits need to be established according to the defined combination of the compounds.

## 4. Conclusions

Breastfeeding is the natural and optimal way of feeding an infant. Current research still focuses on infant formula aiming to more closely resemble the composition and functionality of human milk, with some already comprising probiotics, prebiotics, synbiotics, and postbiotics. Human milk naturally provides these components by delivering HMOs (‘natural prebiotics’) and beneficial bacteria (‘natural probiotics’) and their metabolites (‘natural postbiotics’). In infant formula, these nutritional concepts are provided by different pre- and probiotic mixtures and a mixture of both (synbiotics) and, additionally, by using partly fermented infant formula with beneficial compounds produced by microorganisms that are released from food components or microbial constituents, including non-viable cells (postbiotics). One of the most current examples of such compounds is 3′-galactosyllactose (3′-GL), which is present in human milk and is a natural derivative of milk fermentation. Although such developments may pave the way for future infant formula alternatives for those infants who are not able to be (fully) breastfed, human milk feeding will always remain the unmatched goal for infant nutrition and development as well as provide many benefits for maternal health.

## Figures and Tables

**Table 1:** Current technologies for obtaining commercially available HMOs for application in infant formula (data on 2′FL and LNnT are adapted from Bych et al. [81], data on 3′-GL from Rodriguez-Herrera et al. [12]).

HMO	Technology	Application	Ref
2′-FL and LNnT	Isolation from human milk	For structural identification and fundamental research applications only	[40,82,83,84]
	Chemo-enzymatic synthesis using recombinantly expressed glycosyltransferases matched with nucleotide-activated donor substrates and acceptors	For generating libraries of asymmetrical multi-antennary HMOs for research purposes only	[85]
	Chemical synthesis from L-fucose, D-galactose, N-acetyl-D-glucosamine, and D-lactose, respectively	Prohibitively expensive for large-scale nutrition applications due to complexity, number of reaction steps, limited availability, and high cost of raw materials	[86,87,88,89]
	Coupling of the bacterial homogenates of two or more recombinant microbial cells overexpressing genes for HMO synthesis	For the industrial manufacturing of LNnT and fucosylated oligosaccharides	[90,91]
	Microbial fermentation of engineered *E. coli* strains for fucosylation reaction (complete removal of the strain and all other non-desired biomolecules after fermentation)	Used for both research and viable commercial production with high titers of 2′-FL and LNnT	[92]
3′-GL	Milk fermentation process using *Bifidobacterium breve* C50 and *Streptococcus thermophilus* 065 and providing 3′-GL as a metabolic by-product at levels of ~250 µg/mL	Proprietary fermentation process (Lactofidus^TM^) for large-scale production of fermented infant formula	[12]

**Table 2:** Impact of infant formula fermented with *Bifidobacterium breve* C50 and *Streptococcus thermophilus* 065 on immune and gut parameters.

Infants	Duration (and Start) of Diet	Diet (No. of Infants)	Impact (Fermented vs. Standard Formula Group)	Ref
**Impact on immune parameters**				
Healthy infants	4 m (from birth)	Fermented (11) vs. standard formula (9)	Higher faecal IgA response to polio vaccine	[132]
Healthy infants	5 m (from 4–6 m of age)	Fermented (464) vs. standard formula (449)	Reduced severity of acute diarrhoea (no effect on incidence and duration of diarrhoea episodes and number of hospital admissions)	[133]
Healthy infants	4 m (from birth)	Fermented (30) vs. standard formula (30); HM (30)	Enhanced thymus size (fermented formula group closer to HM)	[134]
Preterm infants (GA < 35 w)	2–5 w (from birth)	Fermented (21) vs. standard formula (31)	Lower faecal calprotectin and higher secretory IgA (no effect on TNF-α)	[135]
Infants at high risk of atopy	12 m (from birth)	Fermented (66) vs. standard formula (63)	Less positive SPT to cow’s milk (no effect on CMA incidence) and lower incidence of digestive and respiratory potentially allergic AEs	[136]
**Impact on gut parameters** (fermented formula ^1^ additionally contained prebiotic scGOS/lcFOS (9:1))				
Healthy infants	From 0–28 d until 17 w of life	Fermented (77) vs. standard formula (86); HM (90)	Softer stool consistency (fermented formula group closer to HM) No differences regarding parent-reported GI (and related) symptoms and investigator-reported AEs (except lower incidence of infantile colic)	[12]
		Subset of 30 infants per study arm	Stools: lower pH, higher levels of acetic acid and sIgA, increased *Bifidobacterium* sp, and decreased *Clostridium difficile* occurrence (gut microbiota composition of fermented formula group closer to HM)	[137]

^1^ Based on a recently developed fermentation process (Lactofidus^TM^) generating bioactive compounds; one of these was 3′-GL at a level of ~250 µg/mL. Abbreviations: AE (adverse event), CMA (cow’s milk allergy), GA (gestational age), GI (gastrointestinal), HM (human milk), IgA (immunoglobulin A), SPT (skin prick test). Reference [137] is a congress abstract and is currently in preparation for full publication.

## Abbreviations

2′-FL	2′-fucosyllactose
3′-GL	3′-galactosyllactose
4’-GL	4’-galactosyllactose
AD	atopic dermatitis
AE	adverse event
ATCC	American Type Culture Collection
DFL	difucosyllactose
DP	degree of polymerisation
Caco	adenocarcinoma of the colon
CBA	chocolate blood agar
CFU	colony forming units
CMA	cow’s milk allergy
DNA	deoxyribonucleic acid
DSM	Dutch State Mines
EFSA	European Food Safety Authority
ESPGHAN	European Society for Paediatric Gastroenterology, Hepatology and Nutrition
FDA	U.S. Food & Drug Administration
FL	fucosyllactoses
FOS	fructo-oligosaccharides
FUT2	fucosyltransferase 2
GOS	galacto-oligosaccharides
GL	galactosyllactose
GRAS	generally recognized as safe
HM	human milk
HMO	human milk oligosaccharide
IF	infant formula
IgE	immunoglobulin E
ISAPP	International Scientific Association for Probiotics and Prebiotics
kDa	kilodalton
LA	Lactobacillus acidophilus
LB	lysogeny broth
LNnT	lacto-N-neotetraose
LNT	lacto-N-tetraose
NCDO	National Collection of Dairy Organism
NCIMB	National Collection of Industrial, Food and Marine Bacteria
NEC	necrotizing enterocolitis
QPS	qualitative presumption of safety
RNA	ribonucleic acid
SCORAD	scoring atopic dermatitis
scGOS/lcFOS (9:1)	short-chain GOS/long-chain FOS (in a ratio of 9:1)
SCFA	short-chain fatty acid
SEM	standard error of the mean
SL	sialyllactose
spp	subspecies
